# Advances in Molecular Profiling and Categorisation of Pancreatic Adenocarcinoma and the Implications for Therapy

**DOI:** 10.3390/cancers10010017

**Published:** 2018-01-12

**Authors:** Rille Pihlak, Jamie M. J. Weaver, Juan W. Valle, Mairéad G. McNamara

**Affiliations:** 1 Division of Cancer Sciences, University of Manchester, Manchester M13 9NT, UK; rille.pihlak@christie.nhs.uk (R.P.); juan.valle@christie.nhs.uk (J.W.V.); 2 Department of Medical Oncology, The Christie NHS Foundation Trust, Wilmslow Road, Manchester M20 4BX, UK; Jamie.Weaver@christie.nhs.uk

**Keywords:** pancreatic adenocarcinoma, mutations, molecular profiling, clinical implications

## Abstract

Pancreatic ductal adenocarcinoma (PDAC) continues to be a disease with poor outcomes and short-lived treatment responses. New information is emerging from genome sequencing identifying potential subgroups based on somatic and germline mutations. A variety of different mutations and mutational signatures have been identified; the driver mutation in around 93% of PDAC is *KRAS*, with other recorded alterations being *SMAD4* and *CDKN2A*. Mutations in the deoxyribonucleic acid (DNA) damage repair pathway have also been investigated in PDAC and multiple clinical trials are ongoing with DNA-damaging agents. Rare mutations in *BRAF* and microsatellite instability (MSI) have been reported in about 1–3% of patients with PDAC, and agents used in other cancers to target these have also shown some promise. Immunotherapy is a developing field, but has failed to demonstrate benefits in PDAC to date. While many trials have failed to improve outcomes in this deadly disease, there is optimism that by developing a better understanding of the translational aspects of this cancer, future informed therapeutic strategies may prove more successful.

## 1. Introduction

Pancreatic ductal adenocarcinoma (PDAC) is one of the deadliest cancers [1] with a five-year overall survival (OS) for all stages of around 8% in the United States (US) [2] and 3% in the United Kingdom (UK) [3]. Even in patients who had potentially curative surgery followed by adjuvant chemotherapy with gemcitabine and capecitabine, the five-year OS was still only 28.8% in the recently reported phase III randomised ESPAC-4 trial [4]. The Phase III ACCORD [5] and MPACT [6] combination chemotherapy trials in patients with advanced PDAC have been the only studies which reported clinically meaningful significant extensions in median OS in the recent decade. Currently, the combination of 5-fluorouracil, oxaliplatin, irinotecan and leucovorin (FOLFIRINOX) from the ACCORD trial has resulted in the longest-reported OS for patients with metastatic PDAC; median OS was 11.1 months compared to 6.8 months with single-agent gemcitabine [5]. Unfortunately, multiple other clinical trials with either chemotherapy combinations or novel agents have failed to demonstrate a significant OS improvement [7,8,9,10]. Due to poor prognosis and very little improvement in survival, PDAC is a major cause of cancer death and it is estimated that it will become the 2nd leading cause of cancer-related death in the US by 2030 [11], being 3rd [2] and 5th [3] currently in the US and UK, respectively. 

It has been reported that around 5–10% of pancreatic cancers arise in the presence of a family history of this diagnosis [12]. Hereditary breast and ovarian cancer (HBOC) [13], Peutz-Jeghers syndrome (PJS), hereditary non-polyposis colorectal carcinoma (HNPCC) [14], familial adenomatous polyposis (FAP) [15], familial atypical multiple mole melanoma (FAMMM) [16] and hereditary pancreatitis [17] have been linked to an increased risk of PDAC, although the numbers are relatively small. 

The largest study reporting germline mutations in patients with PDAC was recently published by Shindo et al. [18]; germline mutations were identified in 3.9% of patients with PDAC. In their cohort of 854 patients, the most prevalent mutations were breast cancer 2 (*BRCA2*), ataxia telangiectasia mutated (*ATM*), breast cancer 1 (*BRCA1*), partner and localiser of BRCA2 (*PALB2*), mutL homolog 1 (MLH1), cyclin-dependent kinase inhibitor 2A (*CDKN2A*) and tumour protein p53 (*TP53*) [18]. 

This review aims to interrogate the novel mutations and signatures identified in PDAC and assess their potential clinical significance for the treatment of patients.

## 2. Genomic Studies in PDAC

Several recent large-scale studies have revolutionised our understanding of PDAC biology and the genome-level drivers of its development. The rate of single nucleotide variants is low at 2.64 mutations per Mb, well below that of cancers such as lung and melanoma that are driven by strong mutagens [19,20].

By analysing the specific trinucleotide context in which single nucleotide variants occur, patterns associated with unique mutagenic processes can be identified [20]. In PDAC, four dominant mutational signatures have been identified—1B, 2, 3, 6. These are associated with age, apolipoprotein B mRNA-editing enzyme, catalytic polypeptide-like (APOBEC) family of cytidine deaminases, BRCA and mismatch repair (MMR) mutations, respectively [21]. By far the most prevalent of these are the ageing signature and APOBEC signature, both seen in almost all cases of PDAC. In contrast, the BRCA and MMR signatures are found in isolated cases, both of which have therapeutic relevance [19,22]. 

Signatures of large structural variation can also be used to classify PDAC. Waddell et al. [19] defined four subclasses based on structural variants (SV) patterns; unstable, stable, locally rearranged and scattered. Importantly, these subtypes have been shown to be independent of cellularity, a known-confounder in PDAC classification. Of therapeutic relevance, the locally rearranged and scattered subtypes demonstrated frequent amplification of known oncogenes, including rare amplifications of potentially druggable kinases such as Fibroblast Growth Factor receptor 1 (*FGFR1*), B-Raf proto-oncogene, serine/threonine kinase (*BRAF*), cyclin-dependent kinase 6 (*CDK6*) and MET proto-oncogene receptor tyrosine kinase (*MET*). As discussed later, the unstable SV signature was also linked to the presence of homologous recombination deficiency which is another potential therapeutic target [23].

Driver gene identification by integrated analysis of copy number changes, structural variants and single nucleotide variants has shown only a relatively small set of core-mutated genes in PDAC, with a long tail of other more infrequently mutated genes. Interestingly, when clustered into pathways, a clearer picture emerges with 10 main pathways commonly targeted. Commonly targeted gene-sets include, cell cycle, deoxyribonucleic acid (DNA) repair, transforming growth factor beta (TGF beta), NOTCH, wingless-type MMTV integration site (WNT), chromatin, SWItch/sucrose non-fermentable (swi/snf), KRAS proto-oncogene, GTPase (KRAS), mitogen-activated protein kinase (MAPK), roundabout guidance receptor-slit guidance ligand (ROBO-SLIT) (axonal guidance) and ribonucleic acid (RNA) processing [24]. Though specific gene mutations are infrequent, many represent potential novel therapeutic targets [25,26]. 

The timing of mutation development has implications for the utility of targeted drug therapy. Targeting mutations that occur early in disease development is preferential, as they are present in all clones and metastatic sites of the disease [27]. In addition, mutations that occur in pre-malignant disease may make excellent targets for arresting disease before invasion and metastatic spread. Pancreatic ductal adenocarcinoma develops from premalignant changes—pancreatic in situ neoplasia 1–3, in a well-recognised, step-wise transition accumulating specific mutations at each stage [28,29,30]. The exact timing of these mutations has recently been challenged [31]. It appears that in some cases, mutations of *TP53*, SMAD family member 4 (*SMAD4*) and *CDKN2A* may occur in a single event. This evolutionary model of cancer described as a “punctuated equilibrium”, has been suggested to underlie the aggressive presentation of PDAC [31]. This early and clonal accumulation of mutations has recently been verified by sequencing of PDAC primaries and multiple metastases in a small cohort of patients demonstrating little genetic variation between tumours from the same patient, independent of metastatic or primary location of the tissue [32].

Though sequencing of the PDAC genome has identified recurrent mutations in multiple pathways the exact function of many of these remains elusive. There has therefore been a focus on sub-grouping PDAC using a more multi-platform approach, including mRNA, microRNA and proteomic studies. Bailey et al. [24] published data on 456 resected PDACs, including whole genome sequencing, deep exome sequencing and transcriptomics. They identified 4 gene-expression based subtypes: squamous, pancreatic progenitor, immunogenic and aberrantly differentiated endocrine exocrine (ADEX) [24]. However, subsequent integrated genomic, transcriptomic and proteomic profiling of 150 resected PDAC specimens, found similar mutations, but identified that the proposed ADEX and immunogenic subtypes from Bailey et al. correlated with low-purity samples in their cohort, that could in turn suggest that the gene expression profiles were derived from non-neoplastic cell contamination [33]. Thus, it appears that at the RNA level, PDAC can be divided broadly into two categories, basal-like/squamous and pancreatic progenitor. Intriguingly, *TP53* mutations and an overall increase in copy-number change numbers were seen in the Basal-like sub type, whilst GATA binding protein 6 (*GATA6*) amplification and GNAS complex locus (*GNAS*) mutations were more prevalent in the classical sub-type (as described by Bailey et al. [24] and Moffitt et al. [34]) [33]. Given this finding, a more thorough analysis of the recently identified structural variant sub-types with the revised mRNA classification will be informative.

## 3. The Role of Different Mutations in PDAC

### 3.1. KRAS Mutations in PDAC

The majority of PDACs are known to harbour mutations in *KRAS* with a prevalence of around 90–95% in most studies [35]. In the most recent and comprehensive study by the cancer genome atlas research network, *KRAS* mutations were identified in 93% of patients with PDAC. In the *KRAS* wild-type tumours, mutations in other *RAS* pathway genes were identified in 60% of cases, demonstrating the central importance of this pathway to PDAC development [33].

Due to the vast majority of PDACs harbouring *KRAS* mutations, multiple clinical trials have tried to target this aberration with novel treatments, but unfortunately all have failed to demonstrate clinically meaningful benefits in OS [36]. Targeting downstream components in the pathway showed promise in phase 1 trials of mitogen-activated protein kinase (MEK) inhibitors, although subsequent trials reported no benefit of MEK inhibition either in monotherapy or in combination with chemotherapy [37,38]. In addition, targeting *KRAS* activation more directly with a farnesyl transferase inhibitor showed no benefit in a large phase III trial [8].

To date, there is no novel agent that has managed to target mutated *KRAS* in PDAC, though given its centrality, it remains a key target. 

### 3.2. DNA Damage Repair Mutations in PDAC

The seminal study of mutational patterns in cancer by Alexandrov et al. [20] showed a unique mutational pattern, signature 3, to be associated with *BRCA1/2* mutations and homologous recombination deficiency in breast, ovarian and pancreatic cancers. This DNA damage repair (DDR) signature was also seen in PDACs in the study by Waddell et al. [19]. In this study, the signature was associated with the unstable structural variant subtype of PDACs, and both somatic and germline mutations of *BRCA1*, *BRCA2* and *PALB2.*

Platinum agents have been shown to be more effective in tumours with deficient DDR, potentially due to their ability to cause DNA strand crosslinking and induce double-strand breaks, which together with *BRCA1/2* mutations, will not be effectively repaired [39]. To date, there hasn’t been a randomised trial confirming this in pancreatic cancers, although data are promising from retrospective cohorts [40,41].

Homologous recombination (HR) deficiency has also been shown to sensitise cancers to poly (ADP-ribose) polymerase (PARP) inhibition in ovarian cancer [42], and, more recently, also in early phase trials in PDAC [43]. Original trials with PARP inhibitors targeted patients with germline mutations of *BRCA* genes [43]. Of late, the focus has shifted to understanding the role of somatic DDR-pathway mutations and homologous recombination deficiency as a signal for PARP inhibition efficacy. In particular, biomarkers of this phenotype are eagerly sought to potentially widen the group of patients that could be treated with these agents [23]. 

A study by Riaz et al. [44] has shown that in a pan-cancer analysis, bi-allelic somatic and germline alterations in multiple DNA repair genes occur across many cancer types, and are associated with genomic features consistent with a deficiency in HR. Interestingly only 45% of bi-allelic mutation cases were in cancers traditionally thought of as hereditary HR deficiency cancer types. This provides a wider population of patients with cancer who may exhibit deficiency in HR characteristics, and thus may allow reclassification of targetable HR deficiency in these tumours. 

Currently, there are multiple clinical trials open for patients with germline-mutated or HR-deficient PDAC treated with either PARP inhibitors alone, or in combination with chemotherapy [45,46]; the results are keenly awaited.

### 3.3. ATM Mutations in PDAC

In one of the earliest PDAC whole genome sequencing studies, Biankin et al. [35] reported that *ATM* aberrations were present in 8% of their samples of 99 resected early PDAC cases. In another study by the same group, *ATM* was also associated with tumours with unstable genome or the BRCA mutational signature [19]. Perkhofer et al. have also shown that ATM deficiency leads to chromosomal instability in PDAC mouse models [47]. As ATM is involved in cellular response to replication stress, and double-strand breaks [48], it is thought that mutations in this gene could also be linked with aberrant DDR or HR deficiency and sensitivity to PARP inhibitors [49]. 

The role of *ATM* in homologous recombination repair and signature 3 was recently questioned in patients with breast cancer as Polak et al. [50] published their work showing that germline pathogenic variants in *ATM* were not associated with a high level of signature 3. This raises a question of whether novel agents targeting DDR and HR deficiency would also work in *ATM*-mutated tumours. There is currently an ongoing trial in patients with PDAC, that defines *BRCA*ness as HR deficient but germline *BRCA* proficient (tumours with somatic *BRCA* mutation, Fanconi anemia gene, *ATM* or BRCA1/BRCA2-containing complex, subunit 5 (*RAD51*) mutations) and is investigating the efficacy of treating these patients with the PARP inhibitor olaparib [51]. 

Defects in ATM could also be compensated through the activity of ataxia telangiectasia And rad3-related protein (ATR), thus indicating potential synthetic lethality interactions between these pathways, as *ATM* mutated tumours would be vulnerable to ATR inhibition [52]. This was also shown in PDAC cell lines [47] and multiple ongoing early phase clinical trials are investigating the *ATM* mutation as a potential biomarker in trials with ATR inhibitors for this reason [53,54]. 

Mutations in *ATM* have also been linked with a more aggressive form of PDAC. Kim et al. reported that in their cohort of 396 resected PDACs, ATM loss correlated with more vascular invasion (63.3%) and metastatic lymph nodes (92.2%) compared to tumours without ATM loss [55]. They also reported decreased OS in patients with ATM loss, but only in those who also had normal TP53 expression. Russell et al. reported that ATM loss in PDAC also correlated with poorer prognosis and less differentiated tumour phenotype in mice and men [56]. Drosos et al. has also demonstrated that ATM deficiency correlated with *KRAS* mutations and promoted highly metastatic PDAC [57]. They also showed that *ATM* loss accelerated *KRAS*-induced carcinogenesis and *ATM* loss increased genomic instability and thus cell lines were more sensitive to DNA damage-inducing agents like radiotherapy [57].

A recent paper by Ayars et al. [58] also showed that ATM-deficient PDAC cells were exquisitely sensitised to radiation, thus illustrating that *ATM-*mutated PDAC could be more sensitive to radiotherapy, and may thus provide more personalised treatment options for these patients in the future.

### 3.4. TP53 Mutations in PDAC

Mutations in *TP53* have been shown to occur in 50–75% [59] of PDAC and in a more recent analysis in around 73% [33]. Morton et al. have shown that after the initiating activating mutation in the *KRAS* gene, the *TP53* mutation drives the rapid progression of KRAS-induced premalignant lesions to PDAC. They also reported that mutation rather than genetic loss of *p53* specifically promotes PDAC metastasis. Weissmueller et al. [60] have shown that promotion of metastasis in mutant p53 cells is induced through platelet-derived growth factor receptor b (PDGFRb), and this in turn could be a prognostic marker and possible target for clinical trials. 

Significant multidrug resistance-associated protein 1 (MRP-1) and B-cell lymphoma 2 (Bcl-2) overexpression due to *TP53* mutations has been linked to gemcitabine resistance in PDAC cell lines [61]. 

Early clinical trials targeting *TP53* in other cancers have shown exciting results. The most promising agents include WEE1 G2 checkpoint kinase (Wee1) inhibitors and APR-246—a small molecule that reactivates mutant *TP53—*both in combination with chemotherapy [62,63]. Currently there are a number of clinical trials ongoing evaluating these agents in p53-mutated gastric, ovarian, lung and other cancers [64,65,66]. To date, there are no clinical trials of these agents specifically targeting PDAC.

### 3.5. SMAD4 Mutations in PDAC 

Mutation and dysregulation of *SMAD4* has been identified in around 30–64% [19,67] of PDACs, either by whole genome sequencing (WGS) or immunohistochemistry. Although this is common in both resected and advanced PDACs, the role of this *SMAD4*-dysregulation is still unknown. 

Many previous studies have reported that *SMAD4* mutations, and loss, might be associated with worse outcomes and patterns of recurrence after PDAC resections [68,69,70,71]. A meta-analysis by Shugang et al. [72] reported that loss of *SMAD4* in patients with PDAC was associated with poorer OS and was a negative prognostic factor. In contrast, a study by Ormanns et al. [67] showed that at least in advanced PDACs, *SMAD4* loss had no impact on OS, and that its presence actually resulted in increases in progression-free survival (PFS) in patients treated with gemcitabine-based chemotherapy. 

Besides the synthetic lethality seen in HR-deficient cancers with PARP inhibitors, collateral lethality was shown to play a role in *SMAD4*-mutated pancreatic cancers. Dey et al. [73] reported that in *SMAD4*-mutated PDAC, the loss of the neighbouring housekeeping gene malic enzyme 2 (*ME2*) in these tumours, created a cancer-specific metabolic vulnerability to Malic Enzyme 3 (ME3) inhibition. They reported that depleting ME3 selectively killed *ME2*-null PDAC cells, and so hypothesised potential new specific targets for novel inhibitors in *SMAD4*-mutated tumours. Currently, there are no such inhibitors on the market, and further research is needed in the collateral lethality approach in PDAC.

### 3.6. CDKN2A Mutations in PDAC

Cell cycle progression through the G1-S phase checkpoint is driven by phosphorylation of the retinoblastoma protein (Rb) by CDK4/6, allowing release of the key transcription factor E2F1. The protein p16, one of the products of the tumour suppressor gene *CDKN2A*, acts to inhibit CDK4/6-cyclin-D complex activation, thus preventing the G1-S phase transition. Familial atypical multiple mole melanoma may be caused by a mutation in *CDKN2A*, and in around 40% of cases the second most commonly observed malignancy in families with the *CDKN2A* mutation is PDAC [74]. 

Varying numbers of *CDKN2A* mutations have been reported in patients with PDAC. Older polymerase chain reaction (PCR)-based studies have reported mutational rates between 5–12% [12,75]. In an Italian cohort study of mainly advanced (67.9% stage III–IV) PDACs, *CDKN2A* mutations were detected in only 5.7% of patients [75]. In another study by Salo-Mullen et al. [12], PCR-based testing in 17 patients identified 2 mutations (11.8%) in *CDKN2A*. In WGS studies, mutations in *CDKN2A* have been found in 35% (11 structural variants and 24 mutations) of resected PDAC samples [19]. Thus, it appears that deeper sequencing and more comprehensive analysis of mutation types identified significantly more aberrations in *CDKN2A* in these tumours.

The predictive and prognostic role of *CDKN2A* mutations in PDAC is largely unknown. A single study has shown that immunohistochemically detected p16 loss correlated with lymphatic invasion and postoperative widespread metastases, together with TP53 and SMAD4 loss [76]. A meta-analysis [72] which evaluated the effects of SMAD4 loss on the outcomes of patients with PDAC did not address the specific role of CDKN2A loss.

Targeted therapy clinical trials in patients with a variety of cancers and mutations in *CDKN2A* are currently ongoing. One study includes ilorasertib [77], an inhibitor of Aurora kinases, vascular endothelial growth factor (VEGF) and platelet-derived growth factor receptor (PDGFRs) and another is investigating the effects of AZD1775, a Wee1 inhibitor [65] in a variety of cancers. Single case reports have also shown good response to palbociclib, an inhibitor of cyclin-dependent kinases 4 and 6 (CDK4/6) in a patient with *CDKN2A*-mutated breast cancer [26] and uterine leiomyosarcoma [78].

Chou et al. [79] have reported that dysregulation of the p16-cyclinD-CDK4/6-RB pathway in PDAC correlates with sensitivity to CDK4/6 inhibitors. Increased total RB or phospho-RB was predictive of response to CDK inhibitors, irrespective of p16 expression or pathway mutations in patient-derived xenograft and cell line models. Therefore, RB protein expression represents a potential new biomarker in PDAC for clinical trials with CDK4/6 inhibitors. 

No site-specific clinical trials in *CDKN2A/CDK4/6*-mutated PDACs have been performed to date, but two clinical trials evaluating the CDK4/6 inhibitor palbociclib are also recruiting patients with PDAC [80,81].

### 3.7. BRAF Mutations in PDAC

In a whole exome sequencing study by Witkiewicz et al. [82], it was shown that mutations in *BRAF* V600 were present in 3% of mainly early stage pancreatic cancers (different histological subtypes) and were mutually exclusive with *KRAS* mutations. The same number of *BRAF* mutations in PDAC were also reported in the most recent study from the cancer genome atlas research network [33].

In patients with *BRAF V600*-mutated advanced melanoma, treatment with the BRAF kinase inhibitor vemurafenib is now the standard of care first or second-line treatment [83]. In a phase II ‘basket’ study of 122 patients with different *BRAF V600*-mutated cancers treated with vemurafenib [71], some positive survival results were reported, including two patients with pancreatic cancer (one of whom had stable disease for seven months and the other progressed and died within one month). Results of ongoing trials for *BRAF V600*-mutated solid tumours are awaited. 

### 3.8. Microsatellite Instability in PDAC

The extent of PDAC risk in hereditary non-polyposis colorectal cancer or Lynch syndrome is still debated, although small numbers of mismatch repair (*MMR*) mutations have been detected in PDAC [12,19]. Humphris et al. [84] recently reported that *MMR* deficiency was found in 1% of their cohort of 385 resected pancreatic cancers samples, and all of these *MMR-*deficient tumours had different somatic inactivations of MutL homolog 1 (*MLH1*) and MutS protein homolog 2 (*MSH2*), without any germline mutations. 

Microsatellite instability as the hypermutable phenotype caused by *MMR* deficiency has been recently shown to predict response to Programmed death-ligand 1 (PDL-1) blockade in solid tumours [22], and has been licenced in the US for any MSI-high cancer [85]. Although MSI is rare in PDAC, there have been promising results in studies of novel treatments with immunotherapy in solid tumours (including PDAC) [22,86], and thus mutational testing for this deficiency in PDAC could be considered. 

## 4. Mutation Burden in PDAC

Next generation sequencing assays and panels are being more widely used by many centres [87,88,89], and with these come new challenges of understanding the results in the context of the tumour types. Many assays are now measuring mutational burden in cancers in the hope that novel treatments with immunotherapy could be indicated for mutational burden high groups [90]. 

In the previously mentioned work by Humphris et al. [84] they reported that out of the 385 resected PDACs, five were hypermutated, containing ≥12 somatic mutations/Mb and another 15 were classified as having a high mutational burden with ∼4–12 mutations/Mb. This classification of mutational burden comes from colorectal cancer studies, where the threshold for hypermutation was 12 mutations/Mb [91]. The 5 hypermutated tumours were all MSI high, and thus could potentially benefit from treatment with immunotherapy, based on the study by Le et al. where mismatch-repair deficiency predicted response of solid tumours to PD-1 blockade [22]. The additional 15 samples in the high mutational burden group showed no evidence of *MMR* deficiency, although did show evidence of HR deficiency in 8/15 samples (as a contributor to the mutational burden). The potential effect of immunotherapy in this high but not hypermutated group is unknown.

The immunogenic subtype of PDAC from the Bailey et al. data [24] also showed upregulation of cytotoxic T-lymphocyte-associated protein 4 (CTLA-4) and PD-1, hypothesising that this subgroup could be treated with novel immunotherapies. Unfortunately, early phase clinical trials with immunotherapy in all patients with PDAC have failed to show response both with CTLA-4 inhibitors and anti-PD-1 therapy [92,93,94]. To the authors’ knowledge, no current clinical trials have specifically looked at targeting the immunogenic subtype of PDAC.

Recently published hypermutation analysis on >81,000 tumours from the Foundation One assay have reported interesting results for PDAC [90]. Similar to previous studies, they reported MSI in pancreatic cancers. However, they also showed that in a proportion of pancreatic cancers, hypermutations could be attributed to tobacco smoke and alkylating agent mutational signatures. Interestingly, alkylating agents are not used in the treatment of pancreatic cancer, thus the presence of this signature is hard to explain. 

Multiple combination immunotherapy clinical trials are currently ongoing in patients with PDAC, looking at combining immunotherapy with either chemotherapy, targeted therapy, another checkpoint inhibitor or radiation (Table 1), and thus the hope of a breakthrough for immunotherapy in treating non-MSI pancreatic cancers continues.

## 5. Other Rare Alterations in PDAC

Additional alterations in the WNT signalling pathway like ring finger protein 43 (RNF43) have been found in 5–10% of patients with PDAC [19,24]. In a recent study by Steinhart et al. [112], it has been shown that mutations in *RNF43* resulted in vulnerability of pancreatic cancer cell lines to WNT inhibition, and thus could be a target for these agents. Currently there is a phase I clinical trial on-going evaluating WNT inhibitors in patients with malignancies dependent on WNT ligands and includes patients with *RNF43*-mutated pancreatic cancer [113]. 

Oncogene amplification is a common mechanism of deregulation in multiple cancer types and is frequently used as a predictive biomarker for targeted therapies [114]. In PDAC, amplification of oncogenes is relatively uncommon in comparison to other gastrointestinal (GI) malignancies, however the locally rearranged and scattered SV subsets of PDACs are enriched for these [19]. In particular, amplifications of Erb-B2 Receptor Tyrosine Kinase 2 (*ERBB2*), *CDK6* and *PIK3CA* are seen in rare cases, all of which may represent potential drug targeting opportunities [114,115,116]. 

## 6. The Feasibility and Challenges of Clinical Molecular Profiling in PDAC

As molecular profiling of all cancers is getting more accessible around the world and different assays are performed in major cancer centres, the actionability of these alterations becomes more and more important. Currently there are no standard-of-care targeted therapies in patients with PDAC, although multiple trials are ongoing. 

In their recent paper, Lowery et al. [117] reported on the feasibility of genomic profiling of PDAC in a real-time clinical setting. In their prospective next generation sequencing (NGS) based assay analysis of tumour tissue and matched normal DNA from 336 patients with PDACs, they reported a median of 45 days between sample collection and the clinical team receiving genomic results. There is no approved targeted therapy in pancreatic cancer, but they did find that 5.5% of patients harboured somatic alterations that are US Food and Drug Administration (FDA)-approved treatment biomarkers in other cancers. These alterations included *BRCA1/2* mutations, *ERBB2* amplifications, *CDK4* amplifications, *BRAF V600E* mutations, ROS proto-oncogene 1 (*ROS-1*) and Anaplastic Lymphoma Receptor Tyrosine Kinase (*ALK1*) involving fusion events. Unfortunately, only three patients (1%) went on to have targeted treatment based on these results; no responses were observed. In addition, six patients with previously known germline *BRCA* mutations received treatment with gemcitabine and cisplatin in combination with a PARP inhibitor, and in total 14 patients with germline-mutated *BRCA* had prolonged responses to platinum-based chemotherapy [117]. 

Similarly, another study by Johns et al. [118] reported that in their cohort of 392 patients with PDAC, 5.1% had somatic mutations that were deemed actionable, but only 1.78% were actioned. Of the 7 actioned, 5 received genetic counselling and 2 received personalised treatment. 

**Figure 1:**
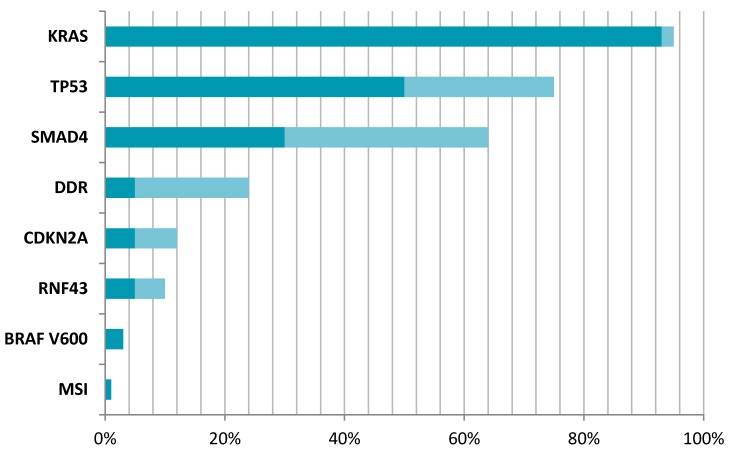
Prevalence of potentially targetable mutations in PDAC. *KRAS—KRAS* proto-oncogene, *GTPase* [33,35]; *TP53—*tumour protein *p53* [59]; *SMAD4—SMAD* Family Member *4* [19,67]; DDR—DNA damage repair pathway mutations [18,19]; *CDKN2A—*cyclin-dependent kinase inhibitor *2A* [12,75]; *RNF43—*ring finger protein *43* [19,24]; *BRAF B-Raf* proto-oncogene, serine/threonine kinase [82]; MSI—microsatellite instability [84]. Dark colour signifies minimal range of mutation reported, while lighter shade signifies maximum range of mutation reported within different references.

Thus, current experience highlights that although actionable alterations in PDAC are rare, it is feasible and may be beneficial in some cases to perform tumour genome analysis (potentially targetable mutations in PDAC are illustrated in Figure 1). Whilst the role for such tests in stratifying patients to receive targeted treatments is currently minimal, there is certainly a role for testing for those changes which might have an impact on genetic counselling of the patient and the family. 

## 7. Discussion

This review aimed to interrogate the current reported data on specific mutations and mutational signatures in patients with PDAC. 

Although the majority of PDACs harbour mutations in *KRAS*, to date there has not been a clinical trial showing efficacy of targeting this mutation in patients with PDAC. Tumours that were previously thought to be *KRAS* wild-type have now been also shown to have mutations in other parts of the RAS pathway indicating that this 5–7% of PDACs are still driven by the same pathway.

The DDR pathway and its potential implications for treatment with platinum-containing chemotherapy regimens and novel targeted therapies such as PARP inhibitors has been most-extensively researched to date. However, prospective clinical evidence on the benefits of platinum agents is currently lacking in PDAC, and is mainly borrowed from other tumour types like ovarian and breast cancers harbouring *BRCA* mutations. Thus, clinical trials on the efficacy of both platinum agents and other DDR-targeting novel agents in this disease subgroup are needed and multiple are on-going.

The role of *SMAD*4 and *CDKN2A* mutations in PDAC is still largely unknown, although there is some evidence that their presence might predict poorer prognosis in patients harbouring these mutations. The concept of novel collateral lethality in *SMAD4*-mutated PDACs has been reported, and clinical trials investigating this phenomenon are eagerly awaited. In addition, novel concepts of measuring RB as a biomarker for CDK4/6 inhibitors and targeting *BRAF* in PDAC are in the early stages of investigation and may give hope for this subgroup of patients.

The use of immunotherapy as a therapeutic option has also been researched in patients with PDAC, with disappointing results in the original single agent trials. Although MSI is rare in PDAC, treatment with the PD-L1 inhibitor pembrolizumab has recently shown promising results in different tumour types harbouring this deficiency, and thus may be an option for a subset of patients with PDAC. In addition, the emerging practice of tumour burden measurement may also help to identify a further subgroup of patients with PDAC who may benefit from this therapeutic approach.

However, to date there are no effective targeted therapies utilised in standard clinical practice for the treatment of patients with PDAC, and so guidelines nor recommendations for regular PDAC profiling for mutations cannot be made. However, novel study designs and initiatives evaluating their clinical relevance as part of a clinical trial are warranted. 

At least two therapeutic development platforms have been established for PDAC: "PRECISION-Promise" in the US and “PRECISION-Panc” in the UK, with the vision being to deliver drug discovery and personalised medicine to this cohort of patients [119]. It is hoped that through coordinated effective preclinical and clinical research, the outcomes for patients with this dismal disease can be substantially enhanced. 

## 8. Conclusions 

Over the last few years, the genetics of PDAC have been revealed through large scale sequencing studies. These studies have clarified oncogenic drivers and their prevalence in PDAC. However, whilst precision medicine has revolutionised the care of many malignancies, no targeted agent has yet been successful in improving outcomes in PDAC. Innovative clinical trials like PRECISION-Panc will be crucial in addressing this deficit.

## Figures and Tables

**Table 1:** Clinical trials registered on Clinicaltrials.gov that are utilising checkpoint inhibitor-based immunotherapy, and are currently recruiting or due to recruit patients with pancreatic ductal adenocarcinoma.

Trial ID	Stage of Disease	Checkpoint Inhibitor	Combined with	Phase	Number of Patients	Countries Involved
NCT03193190 [95]	IV	Atezolizumab	Cobimetinib or PEGPH20 or BL-8040	Ib–II	185	International
NCT01959672 [96]	I–III	Oregovomab	As single agent after chemotherapy and prior to Stereotactic Body Radiation Therapy	II	66	US
NCT03336216 [97]	III–IV	Nivolumab	Cabiralizumab or Cabiralizumab + chemotherapy	II	160	US
NCT03153410 [98]	II *	Pembrolizumab	Cyclophosphamide + GVAX Pancreas Vaccine + IMC-CS4	I	12	US
NCT02734160 [99]	IV	Durvalumab	Galunisertib	Ib	37	International
NCT02648282 [100]	III	Pembrolizumab	Cyclophosphamide + GVAX Pancreas Vaccine + Stereotactic Body Radiation Therapy	II	54	US
NCT03161379 [101]	II *	Nivolumab	Cyclophosphamide + GVAX Pancreas Vaccine + Stereotactic Body Radiation Therapy	II	50	US
NCT02305186 [102]	I–II *	Pembrolizumab	Radiotherapy + capecitabine	Ib–II	56	US
NCT03104439 [103]	IV	Nivolumab and Ipilimumab	Radiotherapy	II	80	US
NCT03190265 [104]	IV	Nivolumab and Ipilimumab	Cyclophosphamide + GVAX Pancreas Vaccine + CRS-207	II	63	US
NCT03006302 [105]	IV	Pembrolizumab	Cyclophosphamide + Epacadostat + CRS-207 + GVAX Pancreas Vaccine	II	70	US
NCT03098550 [106]	III–IV	Nivolumab	Daratumumab	I–II	120	International
NCT03098160 [107]	IV	Ipilimumab	Evofosfamide	I	69	US
NCT02583477 [108]	IV	Durvalumab	Nab-paclitaxel and gemcitabine or AZD5069	Ib–II	19	US, UK
NCT03168139 [109]	IV	Pembrolizumab	Olaptesed pegol	I–II	20	Germany
NCT03329248 [110]	III–IV	Avelumab	ALT-803 + ETBX-011 + GI-4000 + haNK for infusion + bevacizumab + Capecitabine + Cyclophosphamide + Fluorouracil + Leucovorin + nab-Paclitaxel + lovaza + Oxaliplatin + SBRT	I–II	80	US
NCT03080974 [111]	III	Nivolumab	Irreversible Electroporation	II	10	US

* Borderline resectable; US, United States; UK, United Kingdom.
